# Steps Toward Creating A Therapeutic Community for Inpatients Suffering from Chronic Ulcers: Lessons from Allada Buruli Ulcer Treatment Hospital in Benin

**DOI:** 10.1371/journal.pntd.0004602

**Published:** 2016-07-01

**Authors:** Arnaud Setondji Amoussouhoui, Roch Christian Johnson, Ghislain Emmanuel Sopoh, Ines Elvire Agbo, Paulin Aoulou, Jean-Gabin Houezo, Albert Tingbe-Azalou, Micah Boyer, Mark Nichter

**Affiliations:** 1 Faculté des Lettres Arts et Sciences humaines, Université d’Abomey Calavi, Abomey Calavi, Benin; 2 Centre de Diagnostic et de Traitement de l’Ulcère de Buruli, Allada, Benin; 3 Centre Interfacultaire de Formation et de Recherche en Environnement et Développement, Université d’Abomey Calavi, Cotonou, Benin; 4 Institut Régional de Santé Publique, Université d’Abomey Calavi, Ouidah, Benin; 5 Programme National de Lutte contre la Lèpre et l’Ulcère de Buruli, Ministère de la santé, Cotonou, Benin; 6 School of Anthropology, University of Arizona, Tucson, Arizona, United States of America; University of Tennessee, UNITED STATES

## Abstract

**Background:**

Reducing social distance between hospital staff and patients and establishing clear lines of communication is a major challenge when providing in-patient care for people afflicted by Buruli ulcer (BU) and chronic ulcers. Research on hospitals as therapeutic communities is virtually non-existent in Africa and is currently being called for by medical anthropologists working in the field of health service and policy planning. This paper describes a pioneering attempt to establish a therapeutic community for patients suffering from BU and other chronic ulcers requiring long term hospital care in Benin.

**Methods:**

A six-month pilot project was undertaken with the objectives of establishing a therapeutic community and evaluating its impact on practitioner and patient relations. The project was designed and implemented by a team of social scientists working in concert with the current and previous director of a hospital serving patients suffering from advanced stage BU and other chronic ulcers. Qualitative research initially investigated patients’ understanding of their illness and its treatment, identified questions patients had about their hospitalization, and ascertained their level of social support. Newly designed question–answer health education sessions were developed. Following these hospital wide education sessions, open forums were held each week to provide an opportunity for patients and hospital staff to express concerns and render sources of discontent transparent. Patient group representatives then met with hospital staff to problem solve issues in a non-confrontational manner. Psychosocial support for individual patients was provided in a second intervention which took the form of drop-in counseling sessions with social scientists trained to serve as therapy facilitators and culture brokers.

**Results:**

Interviews with patients revealed that most patients had very little information about the identity of their illness and the duration of their treatment. This knowledge gap surprised clinic staff members, who assumed someone had provided this information. Individual counseling and weekly education sessions corrected this information gap and reduced patient concerns about their treatment and the status of their healing process. This led to positive changes in staff–patient interactions. There was widespread consensus among both patients and staff that the quality of communication had increased significantly. Open forums providing an opportunity for patients and staff to air grievances were likewise popular and patient representative meetings resulted in productive problem solving supported by the hospital administration. Some systemic problems, however, remained persistent challenges. Patients with ulcers unrelated to BU questioned why BU patients were receiving preferential treatment, given special medicines, and charged less for their care. The idea of subsidized treatment for one disease and not another was hard to justify, especially given that BU is not contagious.

**Conclusion:**

This pilot project illustrates the basic principles necessary for transforming long term residential hospitals into therapeutic communities. Although the focus of this case study was patients suffering from chronic ulcers, the model presented is relevant for other types of patients with cultural adaptation.

## Author Summary

Little is known about communication patterns and social relations between health staff and long -term patients in African hospitals. An ethnography of a reference hospital treating patients afflicted with Buruli Ulcer (BU) and other chronic ulcers in Benin was conducted. Sources of psychosocial distress and communication patterns compromising quality of care were documented. Based on this research, an intervention was mounted to transform the hospital into a higher functioning therapeutic community. Question: answer education sessions were introduced to provide patients the opportunity to inquire about their illness, it’s treatment and trajectory; weekly open- forums were established to give patients and hospital staff a chance to air grievances; patient representatives met with hospital staff to resolve problems in a non-confrontational manner, and psychosocial support for individual patients was provided through drop-in counseling sessions with social scientists in residence. Patients reported positive changes in the quality of their care and interactions with care providers, care providers reported that the problem solving process instituted was productive, and hospital administrators actively supported efforts to improve social relations and lines of communication. Systemic problems related to perceptions of preferential treatment for BU patients provided subsidized treatment supported by a national program remained contentious.

## Introduction

Providing in-patient care for people afflicted with diseases requiring long-term hospitalization is a major challenge in low-income countries. In these countries, health staff must manage patients with limited resources. At the same time, patients struggle to maintain a positive attitude while far from their families and burdened by concerns about both the progress of their treatment and the welfare of their households during their absence. Patients and hospital staff live and work in close quarters, yet they are often socially distant, their interactions cordial yet primarily focused on disease management tasks. While considerable literature exists in developed countries on the hospital as a social system and on formation of therapeutic communities to care for long-term patients (primarily mental health and substance abuse patients) [1,2], hospital based research on other types of therapeutic communities is sparse, and virtually non-existent for Africa.

This paper describes a pioneering attempt to establish a therapeutic community for patients suffering from Buruli ulcer (BU) and other chronic ulcers requiring long-term care in Benin, West Africa. The hallmark of a hospital-based therapeutic community, as we define it in this paper, is a communication process that invites open dialogue between patients and health staff, patient participation in problem-solving associated with everyday living, ways and means of resolving conflicts that arise, and information exchange that fosters adherence, as distinct from one-sided directives demanding compliance. Our definition of therapeutic community is based on the principle of mutual respect and recognition that respect is only forthcoming when patients and staff better understand the works, responsibilities, challenges, and constraints each faces.

Buruli ulcer (BU) is the third most common mycobacterial disease in the world. A majority of cases are found in West Africa [3]. It is a neglected tropical disease affecting poor rural villagers in several West African countries. Cases diagnosed early can be cured with 56 doses of a combined regimen of intramuscular streptomycin and oral rifampicin. Treatment of advanced cases of BU often requires surgery and long-term residential treatment. During their stay in hospital, a patient’s dressings must be changed daily or at least three times a week, and the patient must undergo physical therapy to prevent disabilities and joint contractures [4,5].

The Allada Buruli Ulcer Treatment Center (CDTUB) is one of the four primary reference centers for BU care in Benin and a recognized center of excellence for clinician training in BU management. The hospital also treats patients suffering from other types of chronic ulcers of various etiologies such as sickle-cell disease, necrotizing fasciitis, and phagedenic or vascular ulcers. Since BU treatment is the primary vocation of the center, BU patients receive subsidized treatment thanks to the government and international NGOs. Patients with advanced BU residing at the hospital require extensive post-operative care. Other chronic ulcer patients have to pay for much of their therapy out of pocket.

When patients suffering from more advanced stages of BU and other chronic ulcers come to hospitals like Allada, they have to adapt to a new way of life in unfamiliar surroundings. They have to learn to get along with other patients who are members of groups they have had little contact with in the past. They then have to cope with the uncertainty of their illness trajectory, the demands of treatment, and the physical discomfort associated with the frequent changing of bandages and physical therapy sessions. For more advanced cases requiring skin grafts, the duration of treatment is uncertain and difficult to predict due to individual variability in wound healing.

Given that the duration of BU treatment is long, and patients are unable to care for themselves, family caretakers are asked to accompany patients and attend to their daily needs such as cooking, washing clothes, and daily assistance. One of the main conditions for being admitted to the hospital is identifying a suitable caretaker from one’s extended kin network. This is often difficult, as removing household members responsible for agricultural operations or child care at home can place the wellbeing of an entire household in peril [6]. In some cases, caretakers come and go, and in other cases they are not able to remain at the hospital and the patient is abandoned [7]. Food is partly provided free of charge for BU patients, but not for their caretakers, and not for patients suffering from other types of chronic ulcers. Although treatment is subsidized for BU patients, there are indirect costs related to hospitalization that can prove burdensome.

## Methods

### Study setting

The CDTUB is located in Allada, a small city of 127, 493 inhabitants located in Benin (West Africa) [8]. It is staffed by four doctors, 18 nurses, eight laboratory technicians, six support staff, three maintenance workers and three drivers. The director of the hospital is a doctor actively engaged in the care of BU patients as well as BU-related research. He is assisted by an administrative staff composed of five secretaries and accountants. The hospital receives approximately 200 new patients a year, out of which around 40 are BU cases. BU patients typically remain in the hospital for 8–18 months, but some remain much longer. Patients in the hospital range from 2 to 70 years of age, with 60% being children. There is an even split between male and female residents in the hospital, with residents divided into nine wards segregated by gender. Caretakers range in age from 9 to 50 years of age, and an overwhelming majority (over 90%) are female.

At the CDTUB, all patients are required to obey rules put in place by the hospital administration to assure a sense of order as well as quality of care. Compliance with hospital policies is mandatory. At the time of the pilot project, patients were treated as passive recipients of care and not provided much knowledge about their disease beyond being told what medications, if any, they were required to take and how to assist in the cleaning and bandaging of their wounds. For patients, their stay at the hospital was a highly liminal experience marked with much apprehension and uncertainty.

### Study design

#### Hospital ethnography

A hospital ethnography was conducted followed by the development and pilot testing of two complementary interventions. A hospital ethnography is a social systems analysis that investigates the ways in which the social organization, administration, therapeutic practices, and interactions between hospital patients, staff and administrators reflect and conflict with social norms, cultural values, and economic contingencies. It begins with a study of the day to day routines, division of labor, and patterned forms of behavior one encounters within hospitals as well as perceptions of quality of care that go beyond clinical guidelines. It then identifies areas of stakeholder concern, tension and conflict, and assesses existing as well as potential processes of problem solving. Hospital ethnographies are a social science contribution to health service research.

In 2013, a three person local social science research team was trained in observational and patient interview methods by an experienced medical anthropologist (MN) who has worked as a therapy facilitator in clinical settings. The team observed patient: staff interactions in Allada Hospital for two months in January-February 2014 and interviewed 42 BU and Non–BU patients residing in the hospital (Table 1) to identify treatment concerns and social relational issues., Illness narratives were first collected to ascertain patients’ health care seeking history, treatment expectations, and understanding of their illness and current treatment. After presenting their illness narrative to a social scientist, patients were asked how they were being treated at the hospital, their interactions with staff and if they were encountering any problems in everyday living. They were also asked about their level of social and economic support while in the hospital. Illness narratives were collected from both adult patients and caretakers of young patients. Interviews were also carried out with 5 staff members to determine what they saw as their scope of work, their communication with patients, and patient compliance problems they encountered on the ward. The data from this research was analyzed and two interventions were developed with the goal of transforming the hospital into a therapeutic community. The interventions were designed by the team of social scientists working in concert with the hospital director and health staff. Intervention options were identified and a SWOT (strengths, weaknesses, opportunities, and threats) analysis conducted to assess the feasibility of each option. The two complementary interventions chosen for piloting were seen as culturally sensitive ways of identifying social tensions in the hospital, eliciting and addressing patients’ concerns, engaging in conflict resolution, and providing social support. The interventions were introduced simultaneously. The combined impact of these interventions on patient: practitioner relations was then assessed thru interviews with both patients and hospital staff.

**Table 1:** Demographic and clinical characteristics of participants in initial qualitative research.

Variables	Participants in residence for BU	Participants in residence for other ulcers	Total of participants
	N = 19	N = 23	N = 42
School level			
No school attendance	10	10	20
Primary	07	09	16
Secondary	02	04	6
Average age (years)	24	35	30
Sex			
Male	09	11	20
Female	10	12	22
Category of participants			
Patient	09	21	30
Caregivers	10	02	12
Average length of stay in the hospital (days)	180	266	223
Characteristics of patient ulcers			
Cat 3 BU	19	00	19
Diabetic Ulcer	00	03	03
Phagedenic Ulcer	00	03	03
Vascular Ulcer	00	02	02
Chronic Ulcer (unspecified)	00	15	15

#### Open forum meetings and an all ward patient committee

The first intervention was designed to provide a space and time for open dialogue about issues causing discontent in the hospital. Considerable research in Africa has pointed to the importance of collective problem-solving in community settings. The open forum was designed to facilitate this process in the hospital.

Weekly meetings were held at the hospital in the evening and attended by both patients and hospital staff. These meetings were designed for two purposes: for continuing education and information exchange, and to provide a collective space for the articulation of grievances. Educational themes selected for weekly meetings included facts about BU and other chronic ulcers, wound and scar care, and health promotion topics related to diet and hygiene. Most of these interactive sessions followed a question-and-answer format modeled after a successful community outreach education program designed by the social science team following a year of qualitative research [9]. Health staff conducted the educational sessions using powerpoint presentations containing evocative photographs and key messages pretested by the social science team for comprehension. Patients were encouraged to ask questions of health staff in attendance. Educational sessions informed patients about health issues so that they could return to their communities as “go to” resource persons for information about BU and wound care.

An open forum followed the education sessions and participants were encouraged to voice concerns about life in the hospital, medical treatment, and conflicts that were brewing. The aims of this open dialogue were not just problem identification and conflict resolution, but also rapport and trust building. The lead social scientist moderated the open forum. Over a seven-month period from March to September 2014, 22 meetings were held, with an average of 60 attendees per meeting including patients, caregivers, and staff. Issues raised at open forum meetings were then referred to a patient committee composed of elected representatives from each of the hospital’s nine wards. Meetings of this committee were held every two weeks. Working together with health staff, the group would try to find solutions to the issues raised in the open forum. The issues and proposed resolutions were then reported to the director of the hospital, who attended some meetings in support of the process. In some cases, conflicts raised led to a review of hospital policy. One or more social scientists attended each meeting and took notes on both the process of communication and attempts at resolution.

#### Private consultation with a social scientist

The second intervention provided patients with an opportunity for an individual consultation with a social scientist as a means of facilitating patient-centered care [10]. There are economic, psychosocial and treatment related issues patients prefer to discuss in private. The lead social scientist established a therapy facilitator role drawing upon lessons learned from medical anthropologists working as cultural brokers in other hospital settings [11].The social science team built on the rapport they had developed with patients while conducting illness narrative interviews [12] and discussing patients level of social and economic support while in the hospital. Therapy facilitation entailed periodic monitoring of patients with interviews attentive to the many “works of illness” patients face during their hospitalization (Table 2) [13,14]. The male and female social science team members maintained an office with an open-door policy in the hospital. Patients and caretakers were encouraged to drop in and discuss emergent problems. Hospital staff occasionally asked a social scientist to talk to a patient who appeared despondent or who was having trouble securing medications necessary for treatment in the case of non-BU patients.

**Table 2:** Key works of illness and treatment for ulcer patients and health staff (adapted from Corbin and Strauss 1985 [13]); Nichter 2005 [14]).

Works of illness: Patients	Brief explanation	Comment: Each kind of work entails different types of effort, consultation, information gathering, accommodation and adaptation
**Pre-hospital illness recognition and self- treatment work**	Symptom recognition as warranting treatment; self- medication	Consultation with others
**Healthcare-seeking work**	Where in pluralistic health care arena should one seek treatment; for how long to evaluate effectiveness	Decision-making–taking into account predisposing and enabling factors, as well as reputation of healer, clinic etc.
**Illness comprehension and treatment work**	What does one know about their disease,	From whom have they gotten information–health staff, other patients?
	*Were they or their family ever told diagnosis by practitioner	Do they feel comfortable asking staff questions?
	*Do they have any idea of how long they will be taking treatment at hospital	
**Preparing to go to the hospital work**	How did household prepare; ramifications of hospitalization on household and livelihood	What support do members of larger social network offer, what kinds of support are requested
**Monitoring healing progress work**	How do they feel healing progress is going; is it what they expected?	Informing family members who inquire
**Pain and sensation management work**	How do patients manage pain and uncomfortable sensations?	Request staff, for medication, self-medication using herbals or medicines bought in market; also work of bearing pain and being stoic
**Compliance/adherence work**	Doing what is requested by staff, managing wound hygiene, physical therapy	Finding resources to do the work expected, following correct procedure
**Subsistence work while in hospital**	Caretaker work, hygiene, food acquisition, etc.	Resources a concern, uncertainty for many; direct and indirect costs
**Social relational work**	Getting along with other patients in the ward and with health staff	Interacting with unfamiliar ethnic groups
**Emotional work**	Maintaining morale during long hospitalization, fighting boredom, fear of abandonment	Managing own emotions plus emotions of household members, managing despondency and depression
**Spiritual work**	Dealing with fears associated with possible etiology, protection from sources of evil	Reduction of fear
**Works of treatment:**	**Brief explanation:**	**Comments:**
**Health staff**		
**Treatment management work**	Adhering to best practices, tailoring treatment to patient	Disease management is primary goal, time pressure due to heavy patient load
**Health education and conceptual translation work**	Explaining to patients how treatment is progressing in terms they can understand	Poor resources exist for this task, often not seen as falling in scope of practice
**Compliance work**	Convincing patient to follow treatment protocol	Patients typically treated as passive
**Trust-building work**	Reassuring patient they are receiving quality care	Favoritism can undermine trust
**Collaboration work**	Collaboration with all stakeholders in hospital from patients to staff to hospital administration	Teamwork essential, social tensions need to be defused
**Making do work**	Making do with resources at hand	Creative problem-solving
**Motivation work**	Fostering and Sustaining motivation	Management needs to foster strong work ethic and provides incentives that reward teamwork

#### Effectiveness assessment

The overall effectiveness of the two complementary interventions was assessed through in-depth open-ended interviews with hospital residents carried out by two of the social scientists who did not play an active role in the implementation of the open-forum intervention. Forty four informants were interviewed about changes in the quality of their care as a result of open-forum meetings and drop-in patient counseling: Fifteen long-term adult BU patients and their caregivers and 29 non-BU chronic ulcer patients and their caregivers were interviewed (Table 3). Key questions focused on changes in lines of communication, levels of knowledge social relations, and problem-solving in the hospital.

**Table 3:** Demographic and clinical characteristics of patients participating in final assessment.

Variables	BU Patients	Non BU Patients	Number of participants
	N = 15	N = 29	N = 44
**School level**			
No school attendance	07	12	19
Primary school	05	13	18
Secondary school	03	04	7
**Average age (years)**	22	33	29
**Sex**			
Male	06	17	23
Female	09	12	21
**Category of participants**			
Patients	07	24	31
Caregivers	08	05	13
**Average length of stay in the hospital (days)**	145	182	169
**Characteristics of patient ulcers**			
Cat 3 BU	15	00	15
Diabetic Ulcers	00	03	03
Phagedenic Ulcer	00	02	02
Vascular Ulcer	00	01	01
Chronic Ulcer (unspecified)	00	24	24

Participants were also asked about the impact of biweekly meetings attended by representatives from the nine patient wards. Questions asked included: did patients and caretakers have a better understanding of hospital rules and regulations as a result of discussions at meetings, were they aware of the issues raised at these meetings, and were any issues/problems resolved in a way that improved the quality of their life? Staff were interviewed about their impressions of the impact of open forum and follow up meetings on patient–staff relations as well as their use of the forum to express their own discontent about issues in the hospital. Social scientists were attentive to staff works of illness (Table 2) and changes in how they view the scope of their work following the interventions.

The lead social scientist recorded observations of staff and patient interactions during the open forum meetings and biweekly follow-up ward representative meetings. All interviews were recorded, transcribed, coded, and entered into Atlas.ti software to facilitate content analysis. Codes were generated from project objectives as well as emergent themes identified from transcript reviews. Data from interviews were triangulated with observational data recorded at weekly meetings. Grounded theory guided data collection and analysis [15,16]. As distinct from a positivist research agenda that tests hypothesis and employs highly structured instruments, a primary aim of grounded theory is to identify participant concerns through more open-ended and semi-structured interviews that reveal both how problems are perceived and described and processes of problem solving.

### Ethical clearance

Ethical approval was obtained from Benin’s National Ethical Committee of Health Research before the start of the research (IRB00006860 N° 148 /MS/DC/SGM/DFRS/CNPERS/SA). Informed consent procedures already in place at Allada hospital were strictly adhered to over the course of the project. All patients and staff interviewed were assured that their opinions would be kept confidential. Patients were assured that information volunteered would in no way affect the quality of their care at the hospital. Patients were also reassured that issues discussed at open forum meetings would not result in negative actions by the staff, and a grievance process was put into place to make sure this did not occur. Oral consent was documented by the presence of witness. The use of oral consent is approved by the ethical review board because many study participants were illiterate. When a participant was under 18 years of age, both the child/adolescent and his/her caretaker were informed about the nature and aim of study before being asked to give consent.

## Results

### Hospital ethnography

#### Initial Qualitative Research: What do patients know about their illness and its treatment and how do they feel they are being treated in the hospital?

Data collected from qualitative interviews at baseline revealed that patients were poorly informed about their disease by health staff. Information was rarely provided to the patient about the type of affliction from which they suffered. At the time of admission to the center, they were told they had a serious health problem that required hospital care and wound dressing. They were then informed that they needed to secure the services of a caretaker to reside with them at the hospital, and told what treatment-related products they would have to pay for out of pocket. Caretakers were required to assist the patient with washing their wounds and changing dressing on a bi-daily basis. Patients were asked to reside in the hospital compound as a clean environment to hasten the healing process and to reduce contact with the world outside the gates of the compound. Notably, 26 of 42 interviewees (61%) were unaware of the type of ulcer for which they were being treated. Levels of awareness did not differ among BU and non-BU cases. When patients and caretakers were asked where they received most of their information about their disease, 26 of 42 (61%) cited more experienced patients in residence at the hospital, not hospital staff. Furthermore, 28 of 42 (67%) interviewees complained that they were given little idea of how the treatment was progressing and how much longer they might have to remain at the hospital. Following admission to the hospital and an initial conversation with staff in which staff sometimes mentioned probable length of stay, these patients and caretakers stated that they received little information about how many more months of hospitalization they were likely to require before being discharged. Patients lived in a liminal state, with great uncertainty about the duration of treatment, and stated that this was highly stress-provoking. For example, one 19-year-old patient expressed his frustration and anger in the following manner:

… It has been over three months since I’ve been here (in the center) with my mother. I was told that after six weeks I should be healed. But I do not know exactly when we will be able to return to my village… The last time my dressing was changed, the nurse examined the wound and reported to me that there was no improvement. But the nurse did not say what this means in terms of my recovery, and the nurse did not tell me what I should do differently. If there is a product to buy, the nurse should tell me…

All patients interviewed at baseline reported that they did not feel comfortable asking hospital staff questions or voicing concerns during routine interactions. Forty-two percent of patients interviewed stated that the only time they would express concern is when they experienced severe pain, and that even then staff were typically unsympathetic. Patients described being rendered docile by busy staff who did not invite questions and referred their complaints to busy doctors who were seen as even more unapproachable than staff. As noted by one patient, a 37-year-old woman who had been in treatment in the center for nine months and was increasingly frustrated by the quality of care:

Health workers do not allow patients to bother them with our concerns. They are difficult to talk to and they shout at us when we complain about pain… there is always pain, it is our main suffering.

Another patient, a 22-year-old BU patient, complained: “I do not approach hospital staff with my problems, because they will just refer you to the doctor. And seeing a doctor is very difficult”.

#### Lack of communication between staff and patients

Follow-up research with health staff identified the pervasive perception that poorly educated and illiterate patients and their caretakers were unable to understand basic information about their disease and its treatment, as well as reasons why they were being asked to follow hospital rules about sanitation, etc. Staff were busy and did not see education or conceptual translation (translation of science into lay terms) as their responsibility. Staff generally defined their role in the hospital as treatment goal-oriented. Further, they did not see eliciting or responding to patient concerns as part of their charge and demanded compliance on the part of patients. Patients perceived staff lack of sensitivity as a sign they cared more about their disease than their person and complained about being treated in a disrespectful manner. One example of this lack of regard related to maintaining ward hygiene. In order to maintain basic levels of hygiene in the wards, hospital staff would pour water over the floors, even when caretakers and patients had left their possessions on the floor, an act that greatly angered ward residents. Health workers justified their behavior, saying that this was the only way they could communicate the importance of hygiene.

These findings greatly surprised the director of the CDTUB, who has been instrumental in initiating community-based BU outreach education efforts in the endemic catchment areas covered by the center. On the basis of these data interventions were developed and piloted in the hospital.

### Interventions

#### Education sessions

Hospital education sessions were modeled after community based BU outreach programs developed by social scientists from the Stop Buruli Consortium following a year of qualitative research in Benin, Cameroon, and Ghana [9]. These sessions followed a question–answer format that was iterative and covered all aspects of BU, wound care, hygiene, and nutrition. All patients and caretakers found these weekly meetings quite informative. Patients felt at ease to ask staff questions during educational sessions and staff did the best they could to answer questions in ways patients could understand. They were assisted by the lead anthropologist, who was skilled in conceptual translation, having participated in community outreach programs for several months. During hospital-based outreach sessions, many of the same issues surfaced, such as: why so many days of medication were required for some kinds of wounds but not others, why some ulcers spread to different parts of the body, and why ulcers were so commonly found on the extremities of the body. Other questions were more specific and related to current illness experience. For example, patients asked questions about pain management, foods to avoid during treatment, the difference between BU and other chronic ulcers that patients were being treated for at the hospital, and whether medicine for BU was good for other kinds of ulcers.

An assessment at the conclusion of the intervention found that patients had far better knowledge about BU and other ulcers than at baseline. Ninety-two percent of interviewees (N = 44, Table 3) were able to respond correctly to questions about the signs of BU and could name at least two key clinical signs. Ninety-seven percent now had adequate knowledge about what kinds of factors did not cause BU as well as possible risk factors. All informants now recognized that BU was caused by a pathogen “worm” (*wevi* in local language) that required at least 56 days of medication to treat and guarantee that all remaining worms had been eliminated from the body. All now recognized that if BU were treated at an early stage, surgery could be avoided. Patients were also better able to distinguish BU from other ulcers, and could identify differences in treatment. There was also increased knowledge about disease progression for BU vs. non-BU ulcers, particularly slow-healing and difficult chronic ulcers arising from diabetes or sickle-cell anemia. Importantly, staff now recognized that patients were able to grasp basic ideas about their disease when presented in culturally appropriate ways [9].

#### Weekly open forum and bi-weekly follow up ward meetings

Following education sessions, an open forum invited discussion of social tensions influencing life in the hospital and issues fostering discontent. For example, complaints surfaced that staff treat patients better when they provide them gifts. As noted by one 45-year-old patient: “If you want hospital staff to respond to a request, then gifts are the way to do it…only then will they give you special attention.”

Giving gifts to reinforce social relationships is a common practice in Benin not limited to hospital contexts. It is used to develop personal relationships and resolve problems within the bureaucracies of the educational, judicial, and legal systems. The problem with giving gifts is that a number of very poor patients living in the hospital are unable to do so. In practice, this creates two tiers of patients in the hospital, which results in discontent. For the poorest patients, giving voice and acknowledgment to their frustration helped diffuse tension around the issue.

The administration quickly came to see patient forums as a useful mechanism for effective exchange and communication that had not been previously available. Health staff would attend the meetings and explain hospital policies and their medical rationale. The real value of the forums, as opposed to other mediums of communication, lay in the possibility of not merely conveying rules to the patients, but in patient-staff negotiation to establish protocols that at once protected patients and were also responsive to their social and psychological needs. Negotiations took place during biweekly ward representative meetings. Requests raised at these meetings were then forwarded on to the hospital director, who examined them and introduced new policies when they appeared reasonable.

One example of a productive exchange concerned patient mobility. Hospital staff had become increasingly frustrated with patients who were leaving the hospital without permission to travel over the weekends, visit local markets, or establish relationships in the larger community. For patients this was a chance to engage in petty entrepreneurial activities to earn much-needed resources as well as visit with relatives, or take a break from the rather monotonous life in the hospital. Although these activities were an important part of the social and economic life of patients, they were also a threat to their healing process and increased the risk of secondary infections. As a first response, the hospital imposed a stringent system of constraints on patient mobility. Patients were confined to the hospital premises and had to request permission to leave hospital grounds. Open-forum discussion identified the issue as a source of discontent and ward meetings established a process whereby hospital policies were altered through a process of negotiation.

The hospital agreed to relax its policy on patient mobility outside the hospital, but made it clear that patients had a responsibility to participate in their healing process. Patients were allowed to leave the hospital, but made aware of the risks to their healing process posed by engaging in different types of activities and exposing their bandages to sources of contamination. Social scientists have observed that patients have not abused their new found freedom and have limited their movement outside the hospital, preferring to give errands to their caretakers whenever possible.

Not all types of discontent aired during open forum were able to be resolved. The most difficult issue that emerged was a systemic problem generic to all linear disease control programs funded by foreign sources of philanthropy that privilege one health problem over others. It was very difficult for patients who have non-BU related chronic ulcers to understand why preferential treatment is offered to cases of BU as distinct from other ulcers. As noted, the care of patients with BU is subsidized through external support offered to the hospital by international NGOs and partners. BU patients do not have to pay for treatment costs and receive a monthly food ration, while other ulcer patients have to pay for treatment and do not receive free food. Non-BU patients, many of whom were unaware of their diagnosis, did not understand this policy and saw existing practices as discriminatory and a display of favoritism. Even when patient diagnosis was clarified, there were patients who felt that preferential treatment by diagnosis was unjust particularly since BU was non-contagious. For example, one male patient aged 32 noted at an open forum: “We do not understand why patients with BU receive free care… and we have to pay for our care… but we all suffer from ulcers, we share the same rooms, and we get the same dressings. As a systemic problem reported by other horizontal programs (diabetes patients complaining about preferential treatment for patients with HIV, for example [17, 18]), there was little that the CDTUB could do given existing funding streams and MOH policy. While the grievance could not be resolved, at least the reasons for the policy were rendered transparent.

One positive outcome of open forum health education sessions was increased staff–patient communication about a key patient concern: pain. In the past, when patients experienced pain or sensations such as itching or burning, they did not report this to staff because such complaints were typically brushed aside. Many patients engaged in self-medication, obtaining medicines from shops, other patients, or healers. Staff complained that this practice often impeded the process of healing and it became a source of tension. Following education sessions that addressed pain, the need to keep wound dressing moist to reduce itching, and how certain sensations constituted a sign of wound closing, patients were more willing to report these symptoms to staff. Patient practices of self-medication also decreased. One informant commented on how increased staff accessibility and the ability to ask questions altered the way he attempted to manage pain:

Before, I had a fear of approaching hospital staff and would not think of knocking on their door… Now it’s easier to approach them and tell them our problems. If, for instance, you experienced pain and did not sleep all night, you can go and tell them and they will assist you. Before these meetings, we did not do that and went and purchased street drugs suggested by friends or other patients… The weekly discussion sessions also helped me understand many things. When the hospital staff is interested in teaching us, this is very good… Male patient, 22 years old

Another positive outcome of educational sessions and open forum discussion was increased patient and caretaker participation in improving hygiene and sanitation in the center. As one nurse noted:

Before the intervention, it was necessary to put pressure on patients and caregivers to involve them in the cleaning of the hospital rooms. Now there is a big improvement. We can say that they have understood the need for good hygiene in the improvement of their health.

#### Patient counseling and therapy facilitation

Social scientists established rapport with patients and caretakers during interviews in which patients’ illness narratives, sources of support, and concerns were explored. The result was patients feeling that they could share personal issues with them, and at the same time necessitated social scientists establishing professional boundaries and a clarification of their role as a therapy facilitator, but not someone who could directly intervene on their behalf. Initially, patient history taking and screening for levels of social support was confined to BU patients, but to offset any feeling of favoritism it was announced at open forum meetings that patients with other types of wounds were welcome to drop in and discuss their problems and concerns with social science staff. Over the six-month period of the intervention, there were over 80 informal discussions of this type. This invitation and social scientists’ receptivity to non-BU patients reduced tensions and jealousies between the two types of patients. Patients felt comfortable bringing issues to social scientists in private that they were not willing to share in public either because they involved conflicts with particular patients or staff members, or entailed personal financial concerns. Employing a works of illness approach to exploring how a patient was coping with different aspects of their illness experience proved useful in deepening and broadening patient assessment.

One of the most significant contributions of individual therapy facilitation meetings was enhanced communication between staff and patients and increased staff feedback about patients’ illness and treatment. A decrease in uncertainty was greatly appreciated by patients and their families. For example, one young man, aged 21, with a chronic ulcer had been growing increasingly frustrated and despondent after having to spend his third Christmas away from his family as an inpatient. He finally learned that his wound was caused by a vascular problem and that further tests might provide additional insight into why his wound was not healing as expected. The tests were not particularly expensive, but well beyond his means. Upon being apprised of this situation health staff contributed to the costs of the tests. Although he did not receive any new treatment as a result of the tests, he was given compression bands. Following an explanation of the underlying cause of his wound, the reason why this kind of wound is difficult to heal, and attention from the staff, his spirits were lifted and he has been reassured that he is getting good care.

Therapy facilitation led to greater compassion shown to patients by staff. For example, in one case a member of the health staff became so frustrated with a patient’s non-compliant behavior (by failing to follow a bandaging procedure in a particular sequence) that he refused to continue with his treatment, referring him to other staff. After a better understanding of the reasons behind his behavior provided by a social scientist, he not only cared for the patient, but did so with genuine empathy about his situation and psychosocial problems. Social scientists documented multiple occasions in which patients who had previously been snubbed or summarily dismissed by health agents were warmly received after they learned more about them following therapy facilitation sessions or during open forums.

Therapy facilitation also entailed dealing with emergent problems that might jeopardize treatment. The hospital has limited funds available to assist the poorest of patients with food and medicine. However, staff do not have the time to make careful assessments about who qualifies, and patients whose financial circumstance worsens during their stay often reveal the gravity of their situation to health staff. Patients in dire need felt comfortable approaching social scientists who carefully assessed whether the problem was short- or long-term. When appropriate, they referred the case to the hospital administration to see if they qualified for support.

Another contribution of therapy facilitation was resolving interpersonal conflicts that were too delicate to air in open forums or be dealt with by ward meetings. In one instance, long-term patients complained of the smell of new patients in the ward, since the necrotization of fresh ulcers produces an unpleasant smell that becomes particularly strong in close quarters. In this case, the problem was presented to the staff member in charge of assigning patients to their beds. An arrangement was made to keep separate wards for new patients and for those whose wounds had largely healed over.

## Discussion

### Challenges to implementing and sustaining a therapeutic community

Three core challenges to establishing a therapeutic community were identified during the pilot project. The first challenge is how to establish an open forum where patients and staff feel comfortable enough to speak their minds without fear of reprisal. If staff feels they are being criticized and that this will have negative impact on their job performance, they will assume a defensive posture. This challenge requires the active support of the hospital director and hospital administration. In the present case, the hospital director let it be known that he viewed the airing of discontent as the first step of a problem solving process that was valued at the hospital. Establishing trust in this process took time and required change on the part of all members of the therapeutic community. By the end of the seven -month pilot project, all stakeholders interviewed had enough trust in the process to feel they could communicate their problems without compromising their position or the quality of care they received.

The second challenge faces social scientists attempting to establish a therapy facilitator/cultural broker role. It is important that they not be seen as the handmaiden of the hospital administration or an advocate for either health staff or patients. Trust demands a neutral position where the charge of the social scientist is to identify, investigate and present all sides of a dispute and to provide in depth understanding of issues affecting administration–staff–patient relations. During the project, there were times when various parties attempted to gain the support of a social scientist in opposition to another. It became important for the social scientist to be clear about what they can and cannot do as part of a process of problem solving. For example, when a patient became destitute because they lost a caretaker or the resources needed for treatment, the social scientists assisted the patient in presenting a case to the administration, but could not be seen as directly solving the resource problem themselves. During the community outreach program that preceded the therapeutic community intervention, the social science team created a resource assessment screening tool to facilitate patient referral to the hospital. The same assessment tool was used in the hospital when an economic crisis was revealed to a social scientist. The screener enabled the case to be systematically presented to the administration after all data necessary to make a decision had been acquired.

A third challenge is sustainability and cost-effectiveness of the social scientist role. The therapeutic community model presented in the study requires the presence of a social scientist and justification for the resources needed to support the position. Based on the results of the pilot study, the Allada hospital administration has decided to employ a social scientist to assist in therapy facilitation and community- based outreach activities, and to secure the services of a psychologist in cases where patients need to be treated for mental health problems requiring medication.

### Conclusion

In this paper, we have described a pioneering attempt to transform an African hospital serving long-term residential patients into a therapeutic community. Although the focus of this case study is BU patients, the model and experience presented here are relevant for many other types of patients. It requires a rethinking of hospital staff–patient relations in concert with the tenets of patient-centered and humanized patient care [19–22] and people-centered health policy [23, 24]. For patients, it addresses their concerns, enhances their sense of well-being, and provides a sense of support and compassion during their long hospital experience. For staff, it leads to greater patient adherence and the resolution of conflicts that can compromise care. In addition, it provides staff as well as patients a forum to articulate their grievances. And for administrators, it provides them with a finger on the pulse of everyday life in the hospital such that tensions can be identified and resolved, policies revisited, and greater transparency provided when necessary.

The pilot project proved to be highly successful as assessed by patients, staff, and administrators. Communication patterns improved, patient uncertainty about the status of wound healing decreased, and patients became far more knowledgeable about their illness. Socially, petty disputes were resolved in a far more amicable fashion, and both patients and staff felt vindicated by expressing discontentment and being heard by others, who could then better understand their position.

The pilot project made use of two distinct but complementary forms of problem solving as a means to establish a therapeutic community in keeping with culturally meaningful modes of conflict resolution in Africa. Much has been written in the anthropological literature about the value of both collective and individual forms of conflict resolution in settings ranging from the settling of social disputes between factions in villages, to processes of divination used to air grievances both past and present [25,26,27]. An open forum both facilitated collective problem solving and enrolled public support for one’s position, serving to establish their moral identity [28]. Individual counseling provided a patient the complementary opportunity to speak to an empathetic witness [29] about difficulties that one would not like to share in public, for reasons ranging from embarrassment to spiritual danger.

Is it feasible to transform African hospitals serving long-term patients into therapeutic communities? We would argue that it is feasible given two conditions. First, hospital administrators need to recognize the utility of building a therapeutic community and be willing to engage in the problem-solving processes outlined in this paper. Second, health social scientists need to receive basic training in health systems analysis and conflict resolution as well as hospital ethnography [30,31] and an anthropological approach to patients’ illness experience attentive to their many “works of illness”. Treating patients as active agents in the hospital will serve as a corrective to paternalistic approaches to patient care that treat them as passive recipients of treatment whose only work is compliance with medical advice [32,33]. Life is far more complicated, and when both patient and staff needs are not met, discontent undermines quality of care.

We would end with one last observation. There is another important way establishing a therapeutic community benefits the hospital. Former satisfied patents are positive sources of information about both the hospital and the community based outreach program it has promoted to identify early category one BU cases. As the old adage goes: the best advertisement is a satisfied customer. This is particularly important in a disease like BU, where the reputation of the hospital is essential to the success of community outreach and the entire BU program. Patients educated in wound care as well as BU re-enter the community as a valuable resource and “go to” person for information about the disease and wound management. In Benin, former patients already play an active role in identifying cases of BU in some communities [34]. Increased patient education and a more positive experience in the hospital increases the likelihood that they will refer chronic ulcer patients to health staff they know and trust.
